# Building resilience: A preliminary exploration of women's perceptions of the use of acupuncture as an adjunct to In Vitro Fertilisation

**DOI:** 10.1186/1472-6882-9-50

**Published:** 2009-12-12

**Authors:** Sheryl de Lacey, Caroline A Smith, Charlotte Paterson

**Affiliations:** 1 School of Nursing & Midwifery, Flinders University, GPO Box 2100, Adelaide SA 5001, Australia; 2 Centre for Complementary Medicine Research, The University of Western Sydney, Locked Bag 1797, Penrith South, NSW 1797, Australia; 3 Institute of Health Service Research, Peninsula Medical School, St Luke's Campus, Heavitree Road, Exeter EX1 2LU, UK

## Abstract

**Background:**

In Vitro Fertilisation (IVF) is now an accepted and effective treatment for infertility, however IVF is acknowledged as contributing to, rather than lessening, the overall psychosocial effects of infertility. Psychological and counselling interventions have previously been widely recommended in parallel with infertility treatments but whilst in many jurisdictions counselling is recommended or mandatory, it may not be widely used. Acupuncture is increasingly used as an adjunct to IVF, in this preliminary study we sought to investigate the experience of infertile women who had used acupuncture to improve their fertility.

**Methods:**

A sample of 20 women was drawn from a cohort of women who had attended for a minimum of four acupuncture sessions in the practices of two acupuncturists in South Australia. Eight women were interviewed using a semi-structured questionnaire. Six had sought acupuncture during IVF treatment and two had begun acupuncture to enhance their fertility and had later progressed to IVF. Descriptive content analysis was employed to analyse the data.

**Results:**

Four major categories of perceptions about acupuncture in relation to reproductive health were identified: (a) Awareness of, and perceived benefits of acupuncture; (b) perceptions of the body and the impact of acupuncture upon it; (c) perceptions of stress and the impact of acupuncture on resilience; and (d) perceptions of the intersection of medical treatment and acupuncture.

**Conclusion:**

This preliminary exploration, whilst confined to a small sample of women, confirms that acupuncture is indeed perceived by infertile women to have an impact to their health. All findings outlined here are reported cautiously because they are limited by the size of the sample. They suggest that further studies of acupuncture as an adjunct to IVF should systematically explore the issues of wellbeing, anxiety, personal and social resilience and women's identity in relation to sexuality and reproduction.

## Background

In Vitro Fertilisation (IVF) is now an accepted and effective treatment for infertility. Although accepted and effective, IVF is acknowledged as contributing to, rather than lessening, the overall psychosocial effects of infertility. Women who have been attempting to fall pregnant for a long period of time or who receive IVF treatment deal with two types of stressors: the chronic stress resulting from the threat of definitive infertility and the loss of future plans for having children, and the acute stress resulting from IVF treatment itself [1,2].

Studies have demonstrated a direct causal link or significant association between stress and reproductive failure [3]. The experience of infertility and the escalating series of interventions involved in diagnosis and treatment culminating in IVF procedures, is widely recognized to represent an unforeseen source of stress for the majority of couples [4]. Women in particular must bear the burden of intervention and inconvenience in IVF processes since her body is the focus of medical intervention and monitoring regardless of the cause of infertility within the couple.

It is recommended that stress is reduced at, or preferably prior to the commencement of IVF treatment [5]. Psychological and counselling interventions have previously been widely recommended in parallel with infertility treatments but whilst in many jurisdictions counselling is recommended or mandatory, it has been reported that less than 25% of women take up these services [4]. A wide range of psychosocial treatments have been implemented yet whilst some were effective in producing positive change, none were effective in influencing pregnancy rate [6].

There has been an increasing interest by women patients and professionals in the use of complementary therapies and medicines (CM) alongside infertility treatments. Three studies in particular have reported on the use of CM by subjects whilst attending for assisted reproduction treatment [7-9]. A recent study conducted in Adelaide, South Australia, for example reported that 60% of subjects had used some form of CM prior to attending a fertility clinic and that the use of CM declined when resurveyed six months later [7]. By contrast, whilst the use of other CM modalities declined over time, the use of acupuncture by women having Assisted Reproductive Technology (ART) increased.

There is some clinical research suggesting acupuncture may increase pregnancy rates when administered as an adjunct to embryo transfer. Eight randomised controlled trials (RCTs) of acupuncture administered as an adjunct on the day of embryo transfer have been included in one of three systematic reviews [10]. Findings and conclusions between systematic reviews differ on the effect of acupuncture and live birth rates, due to variation in their inclusion criteria, the inclusion of new trials in subsequent meta-analyses, and variations in the quality of the acupuncture intervention which may have contributed to clinical heterogeneity. Since publication of the three systematic reviews, two new RCTs have been published [11,12]. Neither trial found a benefit from acupuncture on pregnancy rates, however Domar [2009] found reduced anxiety and increased relaxation and optimism for women receiving acupuncture, although So [2009] found no difference in anxiety between groups.

There are many good reasons to investigate the use of acupuncture in IVF apart from evidence that a woman's chance for pregnancy may be enhanced from acupuncture treatment. For one thing, previous studies of acupuncture involving women dealing with chronic health issues other than infertility have shown that women experienced relief of presenting symptoms but also increases in energy, increase in relaxation and calmness, reduction in the reliance of prescription drugs (such as analgesics), quicker healing from surgery and increased self awareness, sense of wholeness, balance, centredness, well being, increases in self efficacy and overall changes in lives [13,14]. Such effects indicate a reduction of stress that in turn may diminish the number of treatment cycles needed for pregnancy to occur [5]. But further, reducing the number of cycles a woman must undertake to reach her goal of motherhood reduces the overall cost of IVF.

Although in economic terms, the burden of creating embryos is much less for Australians than for those in other countries [15], it is still prohibitive for many couples [16] and there may be many who cannot afford the repetition necessary for successful treatment. Financial pressure has been acknowledged to be an indirect force in patients' decisions regarding treatment [17]. Moreover, whilst the outcomes of IVF have improved with live births per initiated cycle of 20-40% world-wide [18], the overall costs of IVF to the community are significant. The total direct costs of IVF treatment undertaken in 2002 were estimated to be Australian $130.9 million for treatments that involved the transfer of fresh (rather than frozen) embryos, with the average cost of a treatment estimated to be $A6,940.00 [19]. In the United States the cost of an IVF cycle was $12,513, and in Japan $3,956 [20].

Whilst there have been studies of women's experience of acupuncture in relation to chronic health issues other than infertility, there have been no qualitative studies investigating attitudes towards acupuncture among women having IVF. We were interested to understand women's perceptions of acupuncture and their experience of having it during, or prior to having IVF treatment. This paper reports the findings of this preliminary qualitative study, undertaken as a foundation to the planning of the larger mixed methods study in which we plan to combine a clinical trial and patient survey.

## Methods

Ethics approval for this study was obtained from the Research Ethics Committee of the Children, Youth and Women's Health Service in South Australia. Participants were recruited for their shared experience of seeking acupuncture to enhance natural fertility or to complement IVF.

### Sample

For this preliminary study we aimed to achieve ten in-depth interviews with a small homogenous group of women to initially explore our research question. We invited an initial sample of 20 women to participate in the study, drawn randomly from a cohort of women who had attended for a minimum of four acupuncture sessions in the practices of two acupuncturists in South Australia, between January and June 2008.

This initial sample was drawn from a larger group of 300 and selected randomly according to the theoretical categories of 'sought acupuncture during IVF' and 'sought acupuncture for natural fertility enhancement'. Ten women who matched each sampling category were selected for the sample. The 20 women were sent a description of the study and invited to contact an independent researcher (de Lacey). Nine women responded and eight interviews were conducted. Having indicated willingness to participate the ninth woman was unable to be contacted after several attempts. Of the eight, six women had sought acupuncture as an adjunct treatment during Assisted Reproductive Technology, and two women had used acupuncture to enhance their natural fertility and had then progressed to IVF. The demographic characteristics of the sample of women are described in Table 1. Variation in the sample was limited. Although there was some variation in age, duration of infertility and the number of treatments experienced, the sample were similar in that they were largely Australian, middle class, white, married women who had been experiencing infertility for 1-2 years on average. The sample size was restricted by relocation of the acupuncture practice and therefore limited access to the sample. Three patterns of acupuncture use have been observed among women experiencing difficulty with conceiving. We categorized the number of treatments according to this pattern, firstly women having ongoing treatment to assist with conceiving (treatments >10). Secondly, women deciding to use acupuncture to prepare their bodies prior to IVF (treatments 5-9), and lastly a group of women who combine acupuncture and IVF together, usually commencing at the start of an IVF treatment (treatment 1-4)

**Table 1:** Demographic composition of women participants

Characteristic	N = 8
**Age (years)**	
25-30	2
30-35	2
35-40	3
40-45	1

**Marital Status**	
Married	7
Co-habiting	1

**Ethnic background**	
Australian	7
European	0
Scandinavian	1
Asian	0

**Household income ($A per annum)**	
75 - 100,000	5
100 - 125,000	2
>200,000	1

**Length of infertility (years)**	
1-2	6
2-5	2

**Number of IVF treatments**	
1-3	4
4-6	3
7-10	0
>10	1

**Aetiology of infertility**	
Male factor	2
Female factor	2
Combination	1
Unknown	3

**Parity**	
Pregnant	6
Not pregnant	2

**Number of acupuncture treatments**	
1-4	2
5-10	2
10+	4

**Previous use of CMs**	
Yes	4
No	4

### Interviews

A semi-structured interview guide was developed based on three major categories to be discussed with participants: their experience, beliefs and perceptions about (a) the decision to have acupuncture, (b) the process of having acupuncture, and (c) the outcome of having acupuncture. Each question held finer detail. For instance the initial question focused on the history of their fertility problem and the decision to have acupuncture i.e., the onset of infertility, its duration, affect to their life, how they became aware of acupuncture, why they chose to have it and what did they hope the outcome would be. Each interview continued until all three categories were covered and the final question sought any additional information not covered in the categories.

The women were interviewed in their own homes or by telephone. Interview questions were posed and techniques such as open and closed-ended questions specific to their related experience were used to clarify and expand the expression of participant's views [20]. Each interview took approximately an hour and were digitally recorded and transcribed verbatim by a professional transcriber.

### Data analysis

Transcripts of the interviews were de-identified by the use of codes and checked for transcription accuracy. Each researcher then subjected the data to a process of content analysis and coding according to the three interview categories [21]. This was combined with repeated readings of the whole transcript to stay close to the data and maintain the context [22]. In repeated readings the data were checked for similarities and differences in relation to demographic variations such as the outcome of pregnancy and the aetiology of infertility and previous use of CAM. Although the researchers primarily focused on different aspects of the data, due to the low-inference descriptions there was little disparity to be resolved in categorising the codes [23].

### Acupuncture treatment

Acupuncture was administered by two qualified acupuncturists trained in traditional Chinese medicine (TCM), and practicing a patient centered care approach, giving emphasis to the practitioner patient therapeutic relationship, advice on self help and self care, in addition to needling. The acupuncture treatment was based on the findings from a differential diagnosis, and an individualized treatment was administered. For women undergoing an embryo transfer acupuncture treatment was based around evidence based acupuncture treatment protocols used as an adjunct to IVF [10]. Treatment sessions lasted 45 minutes, with mostly bilateral needle retention over 20-30 minutes. Manual stimulation was administered until de qi was obtained, and stimulation techniques included tonification and evens. Treatment frequency was weekly.

## Results

Four major categories of perceptions about acupuncture in relation to reproductive health were identified: (a) Awareness of, and perceived benefits of acupuncture; (b) perceptions of the body and the impact of acupuncture upon it; (c) perceptions of stress and the impact of acupuncture on resilience; and (d) perceptions of the intersection of medical treatment and acupuncture. There were no apparent differences between women's perceptions of acupuncture on demographic variables.

### Perceptions of acupuncture and its appeal

As outlined above, there are now several published studies regarding the use of acupuncture including clinical trials of acupuncture as an adjunct to IVF. As educated, resourceful women the women in this study all described being engaged with the internet in seeking information and support, and seeking strategies to improve their fertility or chances with IVF. They described various processes of learning about acupuncture. One woman had become aware of acupuncture as an adjunct to IVF when visiting the clinic counselor and another described acupuncture as having been recommended by her physician. Both sought further information about it via the internet. Two of the remaining women had used acupuncture before in relation to other problems. Four women had not previously engaged with acupuncture, knew little about it but had become aware of it as a result of internet searches or word of mouth and described how they started to 'come across' articles about acupuncture that raised the possibility that it enhanced fertility.

In their descriptions of the process of acquiring information and perceived benefits of acupuncture whether a recommendation had been received or not, it was apparent that they had all been 'hearing lots about it'. As subject 1 explains, it was the 'helping' element of acupuncture that appealed most.

In an online group of women, a lot of them were talking about it - 'I did acupuncture for this' and you know, some of them sort of went 'I really recommend acupuncture'. Others said 'it didn't work for me 'or whatever, and I just thought 'this sounds interesting'. It just seemed to be a recurring "this helped me" theme in what I was looking at.

### Acupuncture as orthodox therapy

In finding recommendations, and in particular articles about acupuncture, women participants had all been struck by the ways in which acupuncture had been subjected to research. This was described as re-assuring that acupuncture was 'credible therapy' because of its likeness in this way to mainstream medicine, rather than the 'quackery' popularly associated with many complementary therapies. Nonetheless, several women sought validation of its safety and trustworthiness from friends or their doctor before they decided to engage in it.

**Subject 2: **I think the research component of what [the acupuncturist] did and the science behind what she did fitted with my values and kind of made that transition between western and eastern medicine a bit easier for me. She never discounted IVF [but] I think if she's said 'No, you definitely don't need IVF and we can help you and I promise I can help and la, la, la...', then I might have gone off her. She said 'it's not going to be an immediate thing and we don't really now what we can do to help but we'll just try this and this'. I think that what made me think 'Ok, I trust this procedure'.

### Acupuncture as safe to use

Acupuncture was perceived to be more 'natural' than other CMs because it focused on manipulating already existing bodily processes rather than adding substances to alter bodily processes. Although two of the women had engaged in naturopathy previously and all were aware of various herbal medicines and other complementary therapies, they had selected acupuncture to enhance fertility or as an adjunct to IVF treatment. One reason typically advanced for this choice was that herbal remedies required the ingestion of substances that had unknown side effects and interactions. Therefore as non-pharmaceutical medicines, herbal remedies were perceived to hold potential for disrupting the process of ovarian stimulation in IVF or having adverse effects in early pregnancy.

**Subject 1: **I was scared of herbs because I guess I never saw a naturopath as a medical practitioner. I was always scared that they didn't have that, that qualification if you know what I mean or the standing to say they know what they're doing. I think that was the biggest fear, that if I take herbs it's going to stuff up the drugs and then I won't get pregnant.

Women also perceived acupuncture to be more scientific (see above) and also more reliable and predictable.

**Subject 3: **Look it did worry me some of the concoctions that I was being given [when I was seeing a naturopath]. I'm sure that it does people a lot of benefit but I just felt that I was being treated blindly. I found that when I went to see naturopaths that they made lots of promises that they couldn't have known about. Whereas [the acupuncturist] never made promises to fix you. They would just explain that it would be helping your energy flow to those areas. It never made promises.

### Acupuncture as effective - even without pregnancy

By contrast, acupuncture was perceived, even if not effective, to not disrupt reproductive processes. It was therefore a common perception that even if it failed to enhance natural fertility or their chances of becoming pregnant during IVF treatment, it would not diminish them. For subject 4, whose infertility was due to a male factor, acupuncture still represented a means by which she could enhance their chances with no additional risk:

**Subject 4: **I was desperate to get pregnant and happy to try anything. I didn't know if I thought it was going to work or not, but I thought 'it certainly didn't hurt trying it'.

Indeed, the women participants perceived the potential for acupuncture to help them become pregnant to be very high. When asked to rate their perception of its helpfulness in becoming pregnant on a scale of one to ten (1 = not helpful and 10 = very helpful) seven of the eight women rated its potential to help them as being 9-10. The eighth woman rated its helpfulness at 6. When compared, there was no difference in how women perceived acupuncture and the aetiology of their infertility (ie. unexplained, female factor or male factor infertility).

**Subject 1: **I was absolutely convinced that this is what would work. And I don't know why. It was just that I think it was that I was trying something different - hadn't given it a go before. The research was there to say it worked and I probably thought that, 'yep, this is what's going to be the difference between my IVF cycle and say, the next lady's IVF cycle'.

**Subject 3: **Oh I felt very strongly about it. I felt it was really the key. I don't know why. As I said, it could have been something I've invented in my head and it may have been a successful IVF if I had never have done acupuncture but I felt strongly about it.

Six of the eight women participants had become pregnant during IVF and whilst not 100% certain that acupuncture caused the pregnancy, they attributed their success at least in part to the adjunct of acupuncture. But pregnancy was not the only outcome described. Two women remained childless at the time of interview yet perceived acupuncture to have been extremely helpful in assisting them to cope with a negative outcome. While describing several miscarriages finally attributed to a genetic condition blocking the absorption of folic acid, subject 5 explains that although she is aware that acupuncture cannot change that deficiency, it was indeed beneficial:

**Subject 5: **I don't want this to sound like acupuncture hasn't helped me. Because I think it has helped me tremendously. It's not only that it happened [pregnancy] the first time naturally, but also in terms of my mental...how I'm coping and how....yeah, the positivities.

Subject 6 also discussed the role of acupuncture in terms of pregnancy losses and poor treatment outcomes and despite 13 unsuccessful treatments, described her continued use of it in the following way:

I'm past all of it now. Just in myself mentally, I really enjoy it [acupuncture]. I think if you're feeling good, even if its not doing you any good [getting a successful outcome], why stop it?

### Perceptions of the body, of reproductive health and of the physical scope of acupuncture

#### Addressing the 'blocked' body

When asked about how they perceived acupuncture to work, all participants described their bodies as machine-like and acupuncture as being like a tune-up. Typically, women perceived the physical body to have parts that can be dysfunctional and in need of 'oiling' or 'freeing up'. They variously spoke of acupuncture metaphorically as servicing the body, and as clearing blood or energy systems that were 'stuck' so that 'things could get moving' and so that their energy 'flowed better'.

**Subject 3: **If you're uptight and things aren't quite balanced in your body then your messages aren't getting through or whatever. And that's how I imagined acupuncture working in my body: that it was freeing up all these areas where things weren't flowing as they should have been.

#### Improving fertility

The women in the study described physical effects that were perceived to enhance the way their body worked. For example, subject 7 explains how she perceived her body to be more fertile with acupuncture.

**Subject 7: **Every acupuncture session was amazing. It was as if I could feel the blood rushing through my system and I just thought 'well this is doing me so much good' and I'd leave feeling as if my body could do it [get pregnant]. Yeah there were some [treatments] that really made me feel like my body was switching on.

#### Improving vitality

All women described physical effects such as sleeping better, feeling more energetic, more healthy, and more alive.

**Subject 6: **I felt really good after a [acupuncture] treatment. I slept better, woke up fresher, more energized, more confident in myself. I always felt good in myself and I felt healthier, felt alive more.

Despite being given opportunity to explore negative impacts and to express concerns during the interview, the women's perceptions of acupuncture were overwhelmingly positive. Negative effects such as needling discomfort were described as trivial compared with the positive effects to mental wellbeing that were perceived and described.

### Perceptions of the mind, of stress, and of the emotional and cognitive scope of acupuncture

When asked about the effects of acupuncture, all the women described positive effects in terms of their mental health and sense of well-being. This outcome was typically described as being immediate so that they left the acupuncturist with a noticeably improved mood, and as tapering off over the course of the week. This had impact to their personal sense of calm, their sense of self, their ability to cope with day-to-day stresses including medical treatment if they were engaged in it, and their capacity to relate to others and have fulfilling relationships. They variously perceived acupuncture to have been influential in positive medical outcomes or if they were not pregnant, they perceived acupuncture to have helped them optimize their chances through a combined physical (unblocking) and parallel mental soothing and ultimately eased the loss and disappointment. Several different impacts to personal wellbeing were described:

#### Unblocking the mind

Several women described feeling 'peaceful' and less 'busy-minded'.

**Subject 7: **My mind was still really, really clear, probably the clearest it can get.

**Subject 5: **I can start to recall the word peace [when having acupuncture]. I stop worrying and things aren't going around [in my head]. I don't have any need to know about the various things happening and what I have to do next. So I am a lot calmer.

**Subject 3: **It gave me a very warm relaxed feeling, a very kind of centered feeling. And I'd leave the session just feeling okay and balanced and more positive.

#### Mentally stabilising

All women described feeling 'more relaxed', 'balanced', and 'calm'.

**Subject 1: **What I found really surprising was as soon as, the first needle went in, I just felt a sense of relaxation and calm. I wasn't expecting that. It happened every time, yeah, and I suppose I always had this sense of calm and every, after every session regardless of whether I was there after an embryo transfer or just preparing for embryo transfer or whatever, I felt calm and relaxed as I walked out the door. I wasn't expecting that when I went into the whole acupuncture experience.

#### Getting things in perspective

Some women described how the mental effects of acupuncture created a cognitive 'space' characterized by reduced stress, improved mental clarity and increased control that allowed them to view their circumstances more objectively.

**Subject 5: **I'm not sure how much it has been the help of having that possibility to have relaxation. It's like you have that time when she's inserting the needle and you get relaxed. It almost gives you the possibility to go outside yourself and look at things from an external point of view.

#### Increasing tolerance

The ability to cope better was associated with feelings of enhanced well-being and confidence. Women described leaving an acupuncture session feeling refreshed and renewed in terms of energy and tolerance.

**Subject 1: **I just had so much going on in my life at the time [I was having IVF]. Mum was diagnosed with breast cancer and we were struggling with that and struggling with the whole trying to have kids thing. When I walked out of there I just felt like a bit of weight had lifted off my shoulders. It was like, oh I can refocus again. It was like waking up for new day feeling fresh and okay, I can now start to deal with these things again. There were times when I was just so stressed that I felt I just couldn't deal with anything and with the acupuncture, I just felt this sense of calm. It wasn't euphoria, I just felt relaxed and calm.

**Subject 2: **I had a lot of grief related to infertility before acupuncture. I think had I not gone through acupuncture I honestly would have gone into IVF going 'why us? Why us? Why us?' But after going through acupuncture I went into it going 'we are prepared to do what it takes to become parents'.

#### Restoring personal power

All women in this study perceived that they had regained personal control or power that they felt they had lost or relinquished in medical treatment.

**Subject 8: **I do think the acupuncture gave you, oh I don't know, it sort of made me think that you had a little bit of power because you were doing something proactive - without just waiting, having things done to you and then just waiting. You were actually doing something to help besides trying to eat right and all those sort of things.

**Subject 2: **Having acupuncture changed my view of IVF and my lifestyle. After acupuncture everything was working as it should have been so I should have go pregnant, but I wasn't, so therefore to move onto IVF I felt really content that I'd given everything the best shot.

**Subject 7: **That time we did get pregnant. I believe acupuncture helped me relax because I was so stressed by then. It helped me to feel emotionally a bit more in control. I think that definitely helped.

#### Improving relationships

Several women in the study referred to 'flow-on' effects to their social lives brought about though their own changed approach to infertility, personal well-being and therefore communication and interaction with others.

**Subject 7: **I'd say that because I was feeling better within myself, my relationship with my husband improved [husband nodding in agreement] because there were times when I felt so negative, everything I did was negative, everything he did was negative, everything in our house was negative, everything was just negative and nothing could make it any better. And then I'd have the acupuncture and I just felt more positive and I could talk to him like a civilized human being rather than a cross hormonal lady.

**Subject 2: **[My family & peers] would notice a difference when I had acupuncture. They'd call it my 'Zen time'. I was just calm about everything, I wouldn't react so quickly, so strongly to things. So other people would know I'd had acupuncture that day because I'd be a lot more chilled out than normal.

### Perceptions of the intersection of medical treatment and acupuncture

Women in this study described the process of trying to become pregnant, and perceived or diagnosed infertility variously as a harsh and confronting experience. As subject 8 explained, she was engaged in a quest to answer a nagging question of "What else can I do?" The women had all experienced a wish to try something else alongside medical treatment to consolidate their chances of becoming pregnant or to hasten a positive outcome. For the women participants for whom infertility was an unexplained aetiology, there was a possibility that acupuncture would address the unknown factor. Acupuncture was perceived to be an additional therapy that, most importantly, took a different approach to the body and its dysfunction than traditional medicine.

There were various ways that the intersection of acupuncture and reproductive medicine were perceived to combine positively:

#### An adjunct therapy - a different approach to the problem

**Subject 3: **I see it as an adjunct to mainstream treatment or mainstream medicine. I didn't see it as a total cure. But I think acupuncture was beneficial because there's no medical explanation in our case for why things weren't happening. So I think acupuncture offers you another way of managing something.

#### Wholeness

Several women described their sense of being treated as a whole person with physical as well as emotional issues.

**Subject 2: **The first session was a lot of history taking and I think I felt finally that someone was taking a really thorough history, because she goes through everything to do with you and your lifestyle and your health and things like that. She checks all these random things, like your tongue colour and your pulse. That was quite nice to be able to get that stuff out and realise that someone was going to be working with you as a whole system and wouldn't leave anything out.

#### Managing infertility burn-out

Several women described being overcome with emotional exhaustion due to repeated pregnancy attempts, pregnancy losses or disappointments and how they felt they needed to 'throw everything' at the fertility problem

**Subject 1: **I was just so emotionally sick of not being able to do what is supposed to be so easy and natural - to you know, 90, 80 whatever percent of women in the world. I just thought 'I can't do this. I feel like such a failure' and I thought 'I'm willing to try anything' and acupuncture just kept cropping up.

**Subject 7: **When I went on the ovulation induction program I think that psychologically I was in a really bad place then and nothing happened and I think that has a lot to do with that. I just wasn't a very happy person and all this on top of it made it very difficult. [when nothing happened after four rounds of ovulation induction] I was just a mess of tears and I gave up on [clinic] for a while and went to see a naturopath and then a different acupuncturist and they really helped make me ....in a sense got me ready for it all again.

#### Ameliorating medical intervention

All women described the demanding nature of the IVF process and particularly the ways in which it was intensely focused on their physical bodies. As this woman explains, the approach of acupuncture was perceived to truly complement medical intervention in a way that she perceived to provide her with balance and wellness whilst undergoing physical and emotionally demanding medical intervention.

**Subject 7: **Acupuncture could offer me something that the traditional medicine couldn't necessarily offer me and that was just a feeling of wellness and inner health and I felt with the acupuncture, I felt that like my being and my soul were being looked after as well as just making a baby. And I felt that the IVF program is just make a baby, make a baby, make a baby. And although that was my primary aim, I felt that in order for me to be able to make that baby I needed to be looked after emotionally and in my soul as well.

#### Emotional support

Women described a peaceful space that was pleasant and nurturing whilst they underwent acupuncture. In this physical space they described finding emotional space to express their feelings. Some women described crying, others talking freely about their experiences and all described the sense of social support they perceived through the verbal exchange and compassionate care received in acupuncture.

**Subject 2: **There was no room for emotion talk in the fertility specialist's office so the acupuncturist was the only one who'd say to me 'How are you sleeping?' and 'Are you having nightmares?' and then we'd talk about that.

**Subject 4: **I attribute the acupuncture helped me a lot with the kind of emotional stability side. Whether it was the acupuncture or speaking with her, I'm not sure. Everything I would imagine.

**Subject 8: **I thought that she was really good and really listened to you which I found useful because it wasn't always like that at the clinic. When you went to have your blood tests some mornings there were 30 other women in the waiting room. So it's sort of like a production line. But with the acupuncturist, it was easy and she did listen really well and asked how you were feeling about all sorts of things.

**Subject 7: **I really connected with both the acupuncturist who asked me how I was feeling and I could sit there and I could cry so it wasn't just the acupuncture, it was the whole process of it, the whole appointment. By the time I walked out I just felt so much better and so much more refreshed and so much more as if I could cope with whatever the next phase of my IVF treatment was that I was going through.

## Discussion

The findings of this study regarding the experience and perceptions of infertility and acupuncture mirror the findings reported variously by other researchers of acupuncture impact [11,13,14,24-27] in situations of intensive medical care, suggesting that experiences of acupuncture are common and may be directly attributed to the intervention.

In this preliminary study, infertility caused the women to disproportionately focus on their physical bodies and the negative emotions of failure. In particular, IVF treatment intensified this focus to the extent that the women perceived their selves to be 'out of balance'. More than providing a uni-dimensional effect such as reduced anxiety or stress, acupuncture was perceived as acting in multidimensional ways increasing wellbeing, off-setting the negative stress effects of IVF treatment, providing emotional support and giving the women a sense of having gained control - over the treatment, their lives and relationships, and most importantly, their selves. The women described this effect metaphorically as a 'balancing force' and an adjunct to medical intervention. These beneficial effects were reported by all women irrespective of the number of acupuncture treatments administered. Our findings suggest that acupuncture facilitated improvements with coping, and self enhancement.

Acupuncture consists of differing entities contributing to the overall effect of treatment. These entities relate to a dynamic interplay between diagnosis, treatment, needling, co-interventions, patient practitioner engagement, and meaning response from treatment. However, there is a growing opinion that acupuncture cannot be broken down into its constituent parts due to the complex interplay between different entities [28]. Our findings highlight some of the interaction between entities. Firstly, the women described their well-being as increased and that they were better able to cope and interact with significant others. These effects may be related to the treatments administered following a differential Chinese medicine diagnosis, and addressing the women's reproductive potential and emotional distress. In a recent study that compared women having acupuncture during IVF treatment and women who didn't, women in the acupuncture group were shown to have reduced serum cortisol and prolactin levels during the medication phase of IVF treatment with "a trend towards more normal fertile cycle dynamics" [29]. Secondly, the perception of acupuncture in this study may be related to the personal attention and support incorporated in acupuncture treatment. There is little literature describing how acupuncturists work. However, one qualitative study of six acupuncturists in the United Kingdom explored the characterization of the acupuncture treatment process. In this study all practitioners described a pattern of patient centered care based on a therapeutic partnership. Each of the practitioners described the importance of building a therapeutic relationship, individualizing care, and facilitating the active engagement of patients in their own recovery [25]. This approach in acupuncture was characterized by patients as founded on confidence and trust that arose from the acupuncturist's "interest in me as a whole person" as much as recognition of the acupuncturist's expert knowledge [24]. Thirdly. in this preliminary study, emotional and cognitive concerns were largely described as lying unaddressed in mainstream medical approaches despite having, in several cases, repeated contact with health care professionals such as physicians, nurses and, in some cases, counsellors. Acupuncturists aim to develop an attentive presence during the diagnosis and treatment. This presence together with the somato-senory experience from needling may have contributed to the positive experience attributed to acupuncture by women.

The concept of personal and social resilience was an emergent theme in this study. Bonnano [30] described features of resilience identified through psychological studies of people who have suffered trauma and loss. He differentiated resilience from concepts such as recovery and protective psychological mechanisms. Rather, he defined resilience as reflecting the ability to maintain relatively stable, healthy levels of psychological and physical functioning in the face of an isolated and disruptive event. The women who participated in this study described pathways to resilience that related to the components of resilience reported by Bonnano [30] such as: empowerment to effectively influence the outcome of their treatment; self-enhancement through increased self-confidence, cognitive clarity and well being; improved sense of coping and adaptation; and venting of negative feelings that were changed to more positive and optimistic attitudes through holism and social support.

Whilst these components are largely attributed to psychological mechanisms in Bonnano's work, the findings from this exploratory study suggest that other mechanisms unique to acupuncture may be effective in increasing resilience. Previous studies concerning the effects of stress and on fertility or the stress of IVF treatment have shown various forms of psychological counselling make a difference [5,7]. In other studies the net or overall effect of stress and pressure in the experience of infertility and IVF has been described as fragmentation of the self or 'loss of self' [[31,32], de Lacey S: All for Nothing: A postmodern reading of the thwarted search for motherhood through infertility treatment, submitted]. In this study women participants experienced acupuncture as beneficial and perceived that this was because it attended to aspects of their emotions and personhood that were overlooked in conventional medical approaches. Following acupuncture they experienced a sense of balancing or stabilizing of their sense of self (whether they became pregnant as a result of treatment or not) and without access to knowledge about the mechanisms by which acupuncture is effective, they attributed this to the holistic, caring approach of the practitioner.

The limitations of this preliminary study are influenced by the small sample of participants, the narrowness of their demographic profiles, and heterogeneity of the sample with regard to the fertility profile. Although this small sample may limit the extent to which the findings may be reliable, the complexity, depth and amount of data obtained in this qualitative study while indeed small, is by no means too small to obtain a preliminary understanding of the phenomena under investigation. Further, the findings of our study reflect the findings of studies involving non-infertile women accessing acupuncture for different reasons. This suggests an authenticity and trustworthiness of our findings, albeit emerging from a small sample, and indicates that the findings are verifiable. There was some diversity of the fertility profile of women in the sample, and these variables may influence the pregnancy and IVF success rates. However due to the limited collection of clinical data relating to the outcome of an IVF cycle in our study, for example cause of infertility, total dose of FSH fertilisation rate and number of transferred embryos we were unable to examine the relationship between women's experience of acupuncture and reproductive outcomes, or examine the impact of the heterogeneity of women's fertility profile. The findings of this preliminary study are emergent and require further development and research.

## Conclusion

This preliminary exploration, whilst confined to a small sample of women, confirms that acupuncture is indeed perceived by infertile women to have an impact to their health. All findings outlined here are reported cautiously because they are limited by the size of the sample. Nevertheless, the findings of this study reflect the findings of other studies involving non-infertile women accessing acupuncture. They suggest that further clinical studies of acupuncture as an adjunct to IVF should systematically explore the contribution acupuncture can make to wellbeing, anxiety, personal and social resilience and women's identity in relation to sexuality and reproduction. If indeed as suggested here, acupuncture holds the potential to improve a woman's psychological state before during and after IVF treatment, this has important implications for the ways in which it bridges the mind-body gap.

In this small preliminary study the primary purpose was to qualitatively explore women's perceptions of acupuncture in the context of infertility. Future studies will include a mixed method approach in which qualitative data about the embodied experience of acupuncture will be combined with quantitative data regarding IVF treatment, such as the aetiology of infertility, the total dose of FSH administered, the number of follicles aspirated, the number of retrieved ova, and the number and quality of embryos transferred. The combination of different forms of data would be explanatory to the other and together create a rich and deeper understanding of the relationships between acupuncture, the body and IVF outcomes.

## Competing interests

The authors declare that they have no competing interests.

## Authors' contributions

SdL contributed to the conception of the research, undertook the interviews, led the analysis and interpretation of the data and wrote the manuscript. CS conceptualized the research contributed to data analysis, interpretation and commented on the drafts of the manuscript. CP contributed to the methodological design, commented on interpretation of the data and drafts of the manuscript. All authors read and approved the final manuscript.

## Pre-publication history

The pre-publication history for this paper can be accessed here:

http://www.biomedcentral.com/1472-6882/9/50/prepub
